# Dopaminergic reward system: a short integrative review

**DOI:** 10.1186/1755-7682-3-24

**Published:** 2010-10-06

**Authors:** Oscar Arias-Carrión, Maria Stamelou, Eric Murillo-Rodríguez, Manuel Menéndez-González, Ernst Pöppel

**Affiliations:** 1 Human Science Center (FESTO-Program for Applied Knowing). Ludwig-Maximilians-Universität. Munich, Germany; 2 Department of Neurology, Philipps-Universität. Marburg, Germany; 3 Laboratorio de Neurociencias Moleculares e Integrativas. Escuela de Medicina, División Ciencias de la Salud. Universidad Anáhuac Mayab. Mérida, Yucatán. México; 4 Unit of Neurology, Hospital Alvarez-Buylla, Mieres, Spain

## Abstract

Memory is an essential element to adaptive behavior since it allows consolidation of past experience guiding the subject to consider them in future experiences. Among the endogenous molecules that participate in the consolidation of memory, including the drug-seeking reward, considered as a form of learning, is dopamine. This neurotransmitter modulates the activity of specific brain nucleus such as nuclei accumbens, putamen, ventral tegmental area (VTA), among others and synchronizes the activity of these nuclei to establish the neurobiological mechanism to set the hedonic element of learning. We review the experimental evidence that highlights the activity of different brain nuclei modulating the mechanisms whereby dopamine biases memory towards events that are of motivational significance.

## Introduction

Since dopamine (DA) was described as a neurotransmitter in the central nervous system half a century ago [1], its involvement in movement control has long been emphasized due to the association between the amount of striatal DA depletion and motor deficits observed in Parkinson's disease (PD) [2]. Diverse experiments have led to a number of therapeutic interventions to alleviate patients' symptoms, such as L-DOPA therapy [3]. It is known that DA is involved in the neurobiology and symptoms of a myriad of neurological and psychiatric diseases, including schizophrenia and attention deficit hyperactivity disorder, and it is being considered an essential element in the brain reward system and in the action of many drugs with abuse potential [4,5].

Although dopaminergic neurons account for less than 1% of the total neuronal population of the brain[6], they have a profound effect on brain function. For instance, there are modifications of synaptic plasticity as a consequence of learning and memory due to the activity of the metabotropic DA receptors [6,7]. Learning is a change in responsiveness to a particular stimulus whereas memory is the cellular modification that mediates that change. In this regard, recent evidence indicates that DA is involved in reward-related incentive learning [8,9]. However, the mechanism involving DA modulating behavioral choice towards available rewards remains unknown. In this review, we examine the current view of the role of DA in learning and behavioral, with particular regard to reward-seeking behavior.

## Rewarding System and Brain

Rewards are defined as those objects, which we will work to acquire through allocation of time, energy, or effort; that is, any object or goal that we seek [10]. Rewards are crucial for individual and support elementary processes such as drinking, eating and reproduction. The behavioral definition of reward attributes also certain of non-alimentary and nonsexual functions, such as gambling. Rewards engage agents in such diverse behaviors as foraging and trading on stock markets [10].

Due to this requirement, it has been proposed that there exists a single neural system which processes rewards in its different modalities and thereby functions as a common scale through which diverse rewards may be contrasted [11].

Several lines of evidence support the conclusion that the brain's mesencephalic DA system responds to rewards. But, what is the role of DA plays in reward processing? No solid evidence is available about this issue [6,12,13]. However, it has been demonstrated that DA is involved in the hedonic component of reward [6,14]. Several lines of evidence show that the receipt of rewards evokes an increase in DA activity; however numerous conditions exist for which this does not hold. Several hypotheses have been proposed to draw a different mechanism [14,15]. For example, it has been suggested that activity changes in DA neurons encode an error in the prediction of the time and amount of immediate and future rewards (the prediction error hypothesis), therefore, the DA activity is hypothesized to indicate that the immediate or future prospect for reward is better than expected.

## The Mesocorticolimbic Dopamine System

**Figure 1:**
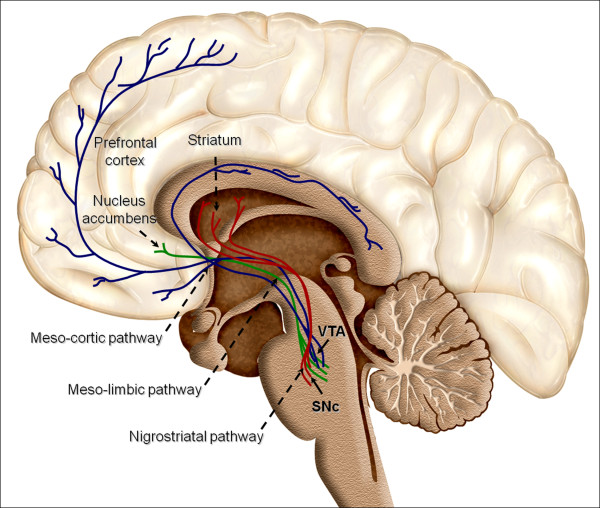
**Overview of reward structures in the human brain**. Dopaminergic neurons are located in the midbrain structures substantia nigra (SNc) and the ventral tegmental area (VTA). Their axons project to the striatum (caudate nucleus, putamen and ventral striatum including nucleus accumbens), the dorsal and ventral prefrontal cortex. Additional brain structures influenced by reward include the supplementary motor area in the frontal lobe, the rhinal cortex in the temporal lobe, the pallidum and subthalamic nucleus in the basal ganglia, and a few others.

In the adult brain, dopaminergic neurons are a heterogeneous group of cells localized in the mesencephalon, diencephalon and the olfactory bulb [6,16]. However, nearly all DA cells reside in the ventral part of the mesencephalon (Figure 1). Mesodiencephalic dopaminergic neurons form substantia nigra pars compacta (SNc), the ventral tegmental area (VTA) and the retrorubral field (RRF). Additionally, the nigrostriatal system, which originates in the SNc and extends its fibers into the caudate-putamen nucleus, plays an essential role in the control of voluntary movement [17,18]. The DA system includes the mesolimbic and mesocortical pathway, which arise from VTA and they have been suggested to modulate emotion-related behavior [14,19,20]. The mesolimbic dopaminergic system include VTA that project mainly to the nucleus accumbens (NAc) as well as the olfactory tubercle innervating the septum, amygdala and hippocampus. On the other hand, the mesocortical dopaminergic system which includes the VTA, extends its fibers in the prefrontal, cingulate and perirhinal cortex. Because of the overlap between these two systems they are often collectively referred to as the mesocorticolimbic system [21,22].

In human brain, there are relatively few neurons in the SNc and VTA (less than 400,000 in the SNc and roughly 5,000 in the VTA [16,23]). Despite that the number of neurons is small, the projections from individual neurons are extensive and hence modulate diverse brain functions. The midbrain dopaminergic neuron is thought to have total axonal length (including collaterals) totaling roughly 74 cm [16] whereas synaptic connections are equally as extensive, with 500,000 terminals common for an individual neuron [16]. In the striatum, approximately 20% of all synapses in the structure [24,25].

From their different nuclei, dopaminergic axons progress medially where they join together and project through the median forebrain bundle (MFB) to the internal capsule [16], then the internal capsule, the axons branch off to form synapses in their target locations [16]. SNc neurons send projections to the caudate and putamen nuclei (striatum), named the nigrostriatal system. Dopaminergic axons originating in the VTA innervates to the ventral part of the striatum, a region named NAc [16].

The diverse physiological actions of DA are mediated by five distinct G protein-coupled receptor subtypes [26,27]. Two D1-like receptor subtypes (D1A-1D and D5) couple to the Gs protein that activate adenylyl cyclase [26,27]. The other receptor subtypes belong to the D2-like subfamily (D2, D3, and D4) and are Gi protein-coupled receptors that inhibit adenylyl cyclase and activate K^+ ^channels [26,27].

The DA receptors have a similar pattern of distribution that dopaminergic fibers [6,28]. For instance, the relative concentration of D1-like receptors compared to D2 receptor is higher in the prefrontal cortex, whereas the concentration of D2-like receptors is elevated in the caudate nucleus, putamen, and nucleus accumbens [26,29]. Importantly, although D1 and D2 receptors have opposite effect at the molecular level, they often act synergistically when more complex outputs are taken into account [30,31].

The neuromolecular mechanism of action of DA is the following: DA is released outside the synaptic cleft [32,33], then it diffuses in the extracellular fluid from which it is slowly cleared as a result of reuptake and metabolism and activates its receptors [34]. One important issue is that DA firing pattern occurs in response to motivationally relevant stimuli [35], it is unlikely that these phasic DA signals influence, to any significant extent, the behavioral response (mediated by fast transmitting pathways) to the same stimulus that triggered them [36,37]. Thus, this neurotransmitter acts as a delayed responding amplifier and modulates behavioral impact [36,37].

## Dopamine Lesions and Disorganized Behaviours

Selective lesioning of DA innervation often reproduces the effects of the lesion itself and disorganizes behavior [38]. The integrative properties of the DA system are probably associated more with direct contributions to cognitive functions at the cortical level, namely in working memory, executive functions and possibly time estimation processes. Since DA brain activity apparently decreases with normal aging, stimulating DA transmission in the elderly could represent a reliable strategy for improving behavioral deficits, as shown in pathological situations such as Parkinson's Disease (PD), where the impairment of DA transmission is evident [39].

## Dopamine and Neurodegenerative Diseases

DA has been associated with neurodegenerative diseases such as PD. It has been demonstrated that a progressive loss of neuromelanin-containing DA neurons in the SNc of the ventral midbrain inducing DA depletion in the striatum, and it has been suggested that this deficit induces motor symptoms associated with PD, including bradykinesia, tremor, rigidity and loss of postural control [40]. In this context, it is interesting to note that the main signs of the pre-frontal syndrome in humans, for example, the diminution in interest in the environment, sensory neglect, distractibility, visuomotor impairment, among others are under DA regulation [41]. Furthermore, negative symptoms of schizophrenia or Alzheimer's disease, also related with DA system [42]. In this regard, a decrease in D1 receptor density in the frontal cortex of schizophrenic patients with negative signs have been shown no change in the striatum [43-45].

## Dopamine and Learning

Instrumental conditioning allows subjects to influence their environment and determine their rate of reward. A general theory is proposed that attributes the origins of human intelligence to an expansion of dopaminergic systems in human cognition [46].

The role of DA on learning and memory has been studied for many years. In this regard, it is known that the D2 receptor agonist bromocriptine modulates working memory performance [47]. Behavioral studies show that DA projections to the striatum and frontal cortex play a central role in mediating the effects of rewards on approach behavior and learning [36]. These results are derived from selective lesions of different components of DA systems, systemic and intracerebral administration of direct and indirect DA receptor agonist and antagonist drugs, electrical self-stimulation, and self-administration of major drugs of abuse, such as cocaine, amphetamine, opiates, alcohol, and nicotine [36,37]. Therefore, more information is required from animal models, where functional studies are possible.

## Dopamine and Reward

Most goal-directed motivation -even the seeking of food or water - is learned [48]. It is largely through selective reinforcement of initially random movements, that the behavior of the neonate comes to be both directed at and motivated by appropriate stimuli in the environment [49]. For the most part, one's motivation is to return to the rewards experienced in the past, and to the cues that mark the way to such rewards. It is primarily through its role in the selective reinforcement of associations between rewards and otherwise neutral stimuli that DA is important for such motivation. Once stimulus-reward associations have been formed, they can remain potent for some time even after the reward has been devalued by the absence of appropriate drive states such as hunger or thirst [48], or because the DA system is blocked [50]. Once a habit has been established, it remains largely autonomous until the conditioned significance of incentive motivational stimuli has been extinguished or devalued through experience. Extinction of the conditioned significance of such stimuli can result from repeated unrewarded trials, repeated trials in the absence of an appropriate drive state, or repeated trials under the influence of neuroleptics [51]. DA appears to be important for learning and memory processes [36].

## The Rewarding System and Addictive Drugs

Over the past 40 years, experimental psychologists have been developing and refining behavioral models of addiction using inventive animal protocols.

Addiction is a neurobiological illness where repetitive substance abuse corrupts the normal circuitry of rewarding and adaptive behaviors causing drug-induced neuroplastic changes. Most findings support that addictive drugs share the common property of enhancing the effect of midbrain DA function, particularly at the level of their terminals in the nucleus accumbens [52,53].

Among the drugs that activate the DA system is cocaine. This compound is a monoamine uptake blocker which binds with greatest affinity to DA transporters which in turn, participate in the mechanism for removal DA from synapses. Blockade of the transporters, therefore, greatly enhances DA's efficacy. It has been indicated that this effect could be the cause of cocaine addiction [54]. Amphetamines activate similar pathway [55,56].

On the other hand, alcohol is believed to affect brain function primarily by enhancing the function of GABA receptors, the primary inhibitory receptors in the brain [57] and reduce the firing rate of neurons in the SNc [58]. Opiates cause a similar release of DA in the striatum [59], both through disinhibition in the VTA and through direct effects on DA terminals [59,60]. Furthermore, blocking opioid receptors in either the VTA or NAc reduces heroin self-administration [61].

Finally, self-administration of nicotine is also blocked by infusion of DA receptor antagonists or by lesion of DA neurons in NAc [62]. The proposal that the dopaminergic system is part of a final pathway for the reinforcing effects of drugs abuse is very appealing and fits in nicely with the literature on brain self-stimulation [63]. Furthermore, chronic exposure to drugs of abuse causes longterm adaptations in cAMP concentrations, tyrosine hydroxylase production, DA expression, receptor coupling to G proteins, and basal firing rate of VTA-DA neurons [64,65]. These mechanisms have been thought to underlie addiction and contribute to relapse to drug taking following periods of abstinence [66-68].

Experimental models to study drug addiction have been developed. For instance, DA transporter KO mice are still capable of developing cocaine addiction [69,70]. This discovery suggested that cocaine's effects would also involving the serotonergic and noradrenanergic transporters [71]. This idea is further supported by the fact that enhanced serotonergic function reduces alcohol self administration [72,73].

## Dopamine and Gambling

A recent study on the other hand, showed faster learning as well as an increase in winning at gambling in response to DA consumption [9]. A simple betting game study by Pessiglione and colleges showed that participants spotted winning strategies at a faster rate if they were given DA in the form of L-DOPA (repetitive). When subjects win a bet, they seem to experience a DA "high" in the form of a reward, which in turn helps them to remember to make the same choice the next time. When the reward for winning was increased through a monetary reward, DA recipients only noticed winning symbols but not the "losing" symbols. These results might explain why L-DOPA treated PD patients become sometimes addicted to gambling [39,74]. DA surges might also explain some of the delusions experienced by people with schizophrenia [41]. Different works have shown that DA is involved in addiction. When people take drugs such as cocaine or amphetamines, they experience artificially induced DA surges which give them the rewarding "high" they crave [22]. The same DA "highs" also occur in people with other addictive behaviors such as gambling, sex and exercise [75]. DA is the brain's mean for reinforcing behavior. Possibly, this work is a system for minimizing prediction errors. Unexpected rewards result in a particularly high amount of DA release and greater learning.

However, recent research finds that while some dopaminergic neurons react in the way expected of reward neurons, others do not and seem to respond in regard to unpredictability [76]. The activity of dopaminergic neurons are thought to be increased by stimuli that predict reward and decreased by stimuli that predict aversive outcomes. Recent work by Matsumoto and Hikosaka challenges this model by asserting that stimuli associated with either rewarding or aversive outcomes increase the activity of dopaminergic neurons in the SNc [76]. This research finds the reward neurons predominate in the ventromedial region in the SNc as well as the VTA. Neurons in these areas project mainly to the ventral striatum and thus might transmit value-related information in regard to reward values. The nonreward neurons are predominate in the dorsolateral area of the SNc which projects to the dorsal striatum and may relate to orienting behavior has been suggested that the difference between these two types of dopaminergic neurons arises from their input: reward-linked ones have input from the basal forebrain while the nonreward-related ones from the lateral habenula [76].

## Conclusions

The past decade has brought an enormous wealth of knowledge on human reward processing using functional brain imaging. Much progress has been made in understanding the neural substrates of human reward processes, but much remains to be learned, and much integration needs to go on among information at the molecular, cellular, systems, and behavioral levels. The pursuit of mechanisms underlying reward has been hampered by the limitations of current animal models and thus requires that basic investigators exchange ideas with those involved in human experimental biology and clinical research. It is clear that neurotransmitters other than DA must play important roles in regulating hedonic states and even in reward-related learning.

Consumption of rewards (e.g., palatable food, mating, cocaine) produces hedonic consequences which initiate learning processes that consolidate liking the rewarding goal. Motivational states such as hunger, sexual arousal, and perhaps early symptoms of drug withdrawal increase the incentive salience of reward-related cues and the reward itself. The greater the hunger, the greater the likelihood that behavioral sequences aimed at obtaining food will be initiated and carried to conclusion despite distractions and obstacles that may arise. Positive reinforcement involves an increase over time in the frequency of behaviors that lead to a reward.

Understanding the neurobiology of the addictive process allows a theoretical psychopharmacological approach for treating addictive disorders, one that takes into account biological interventions aimed at particular stages of the illness.

## Conflict of interests

None of the authors have actual or potential conflict of interest including any financial, personal or other relationships with other people or organizations that could inappropriately influence, or be perceived to influence, our work.

## Authors' contributions

OAC, MS and EP designed, conducted the literature review and drafted most of the manuscript. EMR and MMG performed the literature review and the drafting of the manuscript. All authors were equally involved in reading and approving the final manuscript.
